# Sensitivity of physeal-metaphyseal tractography correlations with height velocity for fractional anisotropy threshold selection

**DOI:** 10.1186/s41747-026-00826-8

**Published:** 2026-10-23

**Authors:** Camilo Perdomo-Luna, Diego Jaramillo, Laura Santos, Sogol Mostoufi-Moab, Ingrid Galvis, Huong Do, Sachin Jambawalikar, Bamidele Kammen, Ola Kvist

**Affiliations:** 1 Department of Radiology, Columbia University Irving Medical Center, New York, NY USA; 2 MicroscopIA Research Group, MicroscopIA, Bogotá, Colombia; 3 Department of Radiology, Hospital for Special Surgery, New York, NY USA; 4 The Children’s Hospital of Philadelphia, Philadelphia, PA USA; 5 UCSF Benioff Children’s Hospital, Oakland, CA USA; 6 UCSF Department of Radiology & Biomedical imaging, San Francisco, CA USA; 7 Department of Children’s and Women’s Health, Karolinska Institute, Stockholm, Sweden

**Keywords:** Anisotropy, Cartilage, Diffusion tensor imaging, Growth plate, Magnetic resonance imaging

## Abstract

**Objective:**

Diffusion tensor imaging (DTI) tractography of the physeal–metaphyseal complex (PMC) provides insights into skeletal maturation. Fractional anisotropy (FA) minimal and maximal thresholds of 0.15 and 1.0, respectively, are commonly used to reconstruct tracts; these values were developed for highly organized tissues and may exclude the less organized hypertrophic and transitional zones that drive growth. Whether lowering the FA thresholds better captures growth-sensitive features and correlates with annualized height velocity is unknown.

**Material and methods:**

Forty-five healthy adolescents (20 boys; 12–17 years) underwent 3 T DTI of the distal femoral PMC. Deterministic tractography was performed using nine previously untested FA threshold combinations (FA_min: 0.05, 0.10, 0.15 paired with FA_max: 0.40, 0.50, 0.60) and a conventional threshold (0.15–1.0). Partial Pearson correlations between extracted metrics (tract FA, apparent diffusion coefficient [ADC], volume) and annualized height velocity were computed, controlling for Tanner stage.

**Results:**

Tract ADC showed strong positive correlations with height velocity across all thresholds (*r* = 0.55–0.63). Tract FA showed consistent negative correlations (*r* = -0.38 to -0.45). Tract volume correlations were threshold-dependent: positive at lower FA_min thresholds (*r* = 0.33–0.37, at FA_min = 0.05) but attenuated or reversed at higher FA_min thresholds (*r* = -0.26, at 0.15–1.0).

**Conclusion:**

Tract ADC and FA are robust metrics of growth plate activity, insensitive to FA threshold selection. Tract volume associations with height velocity are threshold-dependent, with lower FA_min thresholds capturing growth-sensitive PMC tissue and lower FA_max excluding inactive PMC tissues. These findings underscore the importance of standardized tractography parameters in growth plate DTI studies.

**Key Points:**

***Question*** How can we prevent conventional DTI FA thresholds from underestimating growth-sensitive tract volumes in the pediatric PMC?

***Findings*** Lower FA thresholds accurately capture tract volume correlations with height velocity, while tract ADC and FA correlate reliably across all thresholds.

***Relevance statement*** Optimizing FA thresholds in physeal-metaphyseal-complex DTI improves microstructural imaging correlations with height velocity, ensures reproducible data in pediatric skeletal growth studies, enhances noninvasive skeletal growth monitoring in adolescents, and potentially supplements traditional bone-age radiographs.

**Graphical Abstract:**

**Figure:**
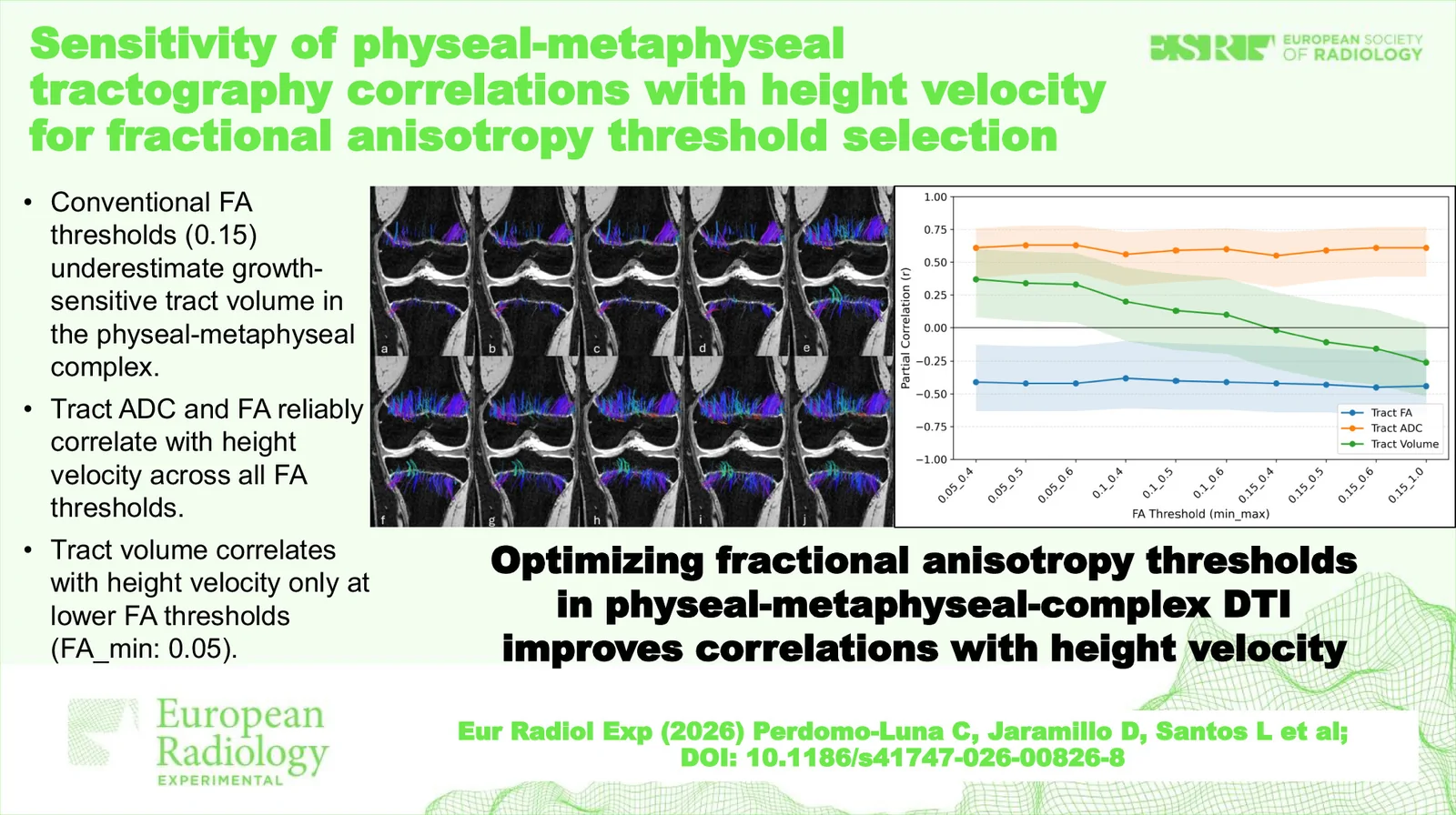

## Background

Diffusion tensor imaging (DTI) is an advanced imaging method used to characterize biological tissues by measuring the diffusion of water molecules. This technique yields several key DTI metrics that provide complementary information. Fractional Anisotropy (FA) quantifies the directionality of water diffusion. Apparent Diffusion Coefficient (ADC) reflects the average rate of water diffusion [1–3].

These DTI metrics provide the foundation for tractography, a computational technique used to reconstruct microstructural pathways and reflect microstructural organization, potentially serving as a surrogate marker of growth-related activity in the physeal-metaphyseal-complex (PMC). Of the PMC, tractography generates its own distinct metrics: tract count, tract length, and the composite of those, tract volume. The reconstruction process critically relies on an FA threshold to determine which voxels are included. The traditional FA thresholds most used in the literature for PMC tractography are minimum (FA min) and maximum (FA max) values of 0.15 and 1.0, respectively. These values have been consistently applied in studies characterizing the columnar structure of the PMC and its changes with age, height velocity, and skeletal location, as demonstrated in both pilot and larger cohort studies. The rationale for this threshold is to balance sensitivity to the highly organized, anisotropic PMC while minimizing the inclusion of noise or isotropic tissue [1, 4–8].

The selection of FA thresholds significantly alters the reconstruction of tract number, length, and volume, reflecting a trade-off in which lower thresholds may capture subtle or less organized microstructural features at the potential cost of reduced specificity and increased false positives. While this optimization challenge has been documented in musculoskeletal DTI of muscle, its specific impact on the sensitivity of PMC tractography to biological growth velocity has not been evaluated [9, 10]. Establishing these optimized, standardized parameters is a prerequisite for the multi-center reproducibility required for clinical implementation.

Our study aimed to evaluate the impact of varying the FA thresholds on PMC DTI and tractography metrics and their correlation with height velocity (height increase over one year), the standard measure used in studies of skeletal growth.

## Methods

### Participants

This study is a methodological sub-analysis of a prospective cohort study conducted by two tertiary pediatric centers, with approval from their respective institutional review boards. All procedures adhered to the Health Insurance Portability and Accountability Act. All parents or legal guardians provided informed consent, and children provided assent.

Participants were recruited from general pediatric practices in two urban cities in the northeastern United States from July 2020 to July 2022. Because of the 12-month interval between visits, some participants turned 17 by the follow up visits; the age range across both visits was therefore 12-17 years of age. Participants were excluded if they had a history of cancer, systemic illness, or medications impacting growth, or if they had metallic hardware precluding magnetic resonance imaging (MRI). At each institution, dedicated research staff trained by pediatric endocrinologists measured participants’ height and weight at baseline and at a 12-month follow-up visit. Weight was measured using a Scale-Tronix (Skaneateles Falls, New York, United States) standing patient scale, while height was measured using a stadiometer from Holtain Limited (Crymych, Dyfed, United Kingdom). Participants performed self-assessment using validated, gender-specific schematic line drawings (Tanner stages I–V), which have been shown to correlate strongly with physician-assessed pubertal status in adolescent research cohorts [11]. While clinical examination by a pediatrician remains the reference standard, self-assessment is a widely accepted proxy in research settings where physical examination of healthy volunteers is impractical.

### MRI acquisition

All participants were imaged twice at a yearly interval. DTI of the left knee was obtained at 3 T using a fat-suppressed single-shot spin-echo echo-planar sequence with a total scan time of 5:21 min:s. The left knee was selected to represent the non-dominant limb for the majority of the cohort, given that right-foot dominance is reported in approximately 60–80% of the population [12], and to maintain consistency with established quantitative imaging protocols for the pediatric physis. Imaging was performed with 20 non-collinear directions; *b*-values of 0 and 600 sec/mm^2^, in-plane resolution 2.0 × 2.0 mm^2^, 3.0-mm section thickness (voxel 12 mm^3^), and no intersection gap. The 25.6-cm field of view included the PMC regions in the femur and tibia. A total of 45 slices were acquired in the sagittal plane, covering the entire PMC.

One center used a 3-T Signa Premier unit (GE Medical Systems, Waukesha, WI, USA) with an 18-channel knee coil (Quality Electrodynamics, Mayfield Village, OH, USA). Parameters at this center were: repetition time/echo time 3,000/51.7 msec; bandwidth 1,953.12 Hz/pixel; parallel imaging factor 2; signal averages 5; matrix 128 × 90; field of view 25.6 × 18 cm^2^; and a slice-selective gradient reversal fat suppression. The second center used a 3-T Magnetom Prisma unit (Siemens Healthineers, Erlangen, Germany) with a 15-channel knee coil (Quality Electrodynamics, Mayfield Village, OH, USA). Parameters at this center were: repetition time/echo time 7,100/82 msec; bandwidth 1,446 Hz/pixel; parallel imaging factor 2; signal averages 2; matrix 128 × 90; field of view 25.6 × 18 cm^2^; and frequency-selective fat suppression with spectral adiabatic inversion recovery–SPAIR. Despite the use of different hardware platforms, sequence parameters—including voxel size, *b*-values, and diffusion directions—were harmonized across sites to ensure technical consistency in the calculated DTI metrics. A prior comparison of results in patients scanned with both platforms using Bland-Altman plots and intraclass correlation revealed high reproducibility across platforms [13]. To minimize inter-site variability, key parameters most directly affecting diffusion quantification, including voxel size, *b*-values, diffusion direction scheme, and spatial coverage, were harmonized across sites where feasible. Due to hardware and vendor-specific constraints, repetition times and echo times were optimized independently for each platform (GE: 3,000/51.7 ms; Siemens: 7,100/82 ms) rather than perfectly matched, ensuring robust signal-to-noise ratios while maintaining quantifiable diffusion metrics. The GE platform and protocol used here are identical to those validated in our prior work [13].

### ROI definition

**Fig. 1:**
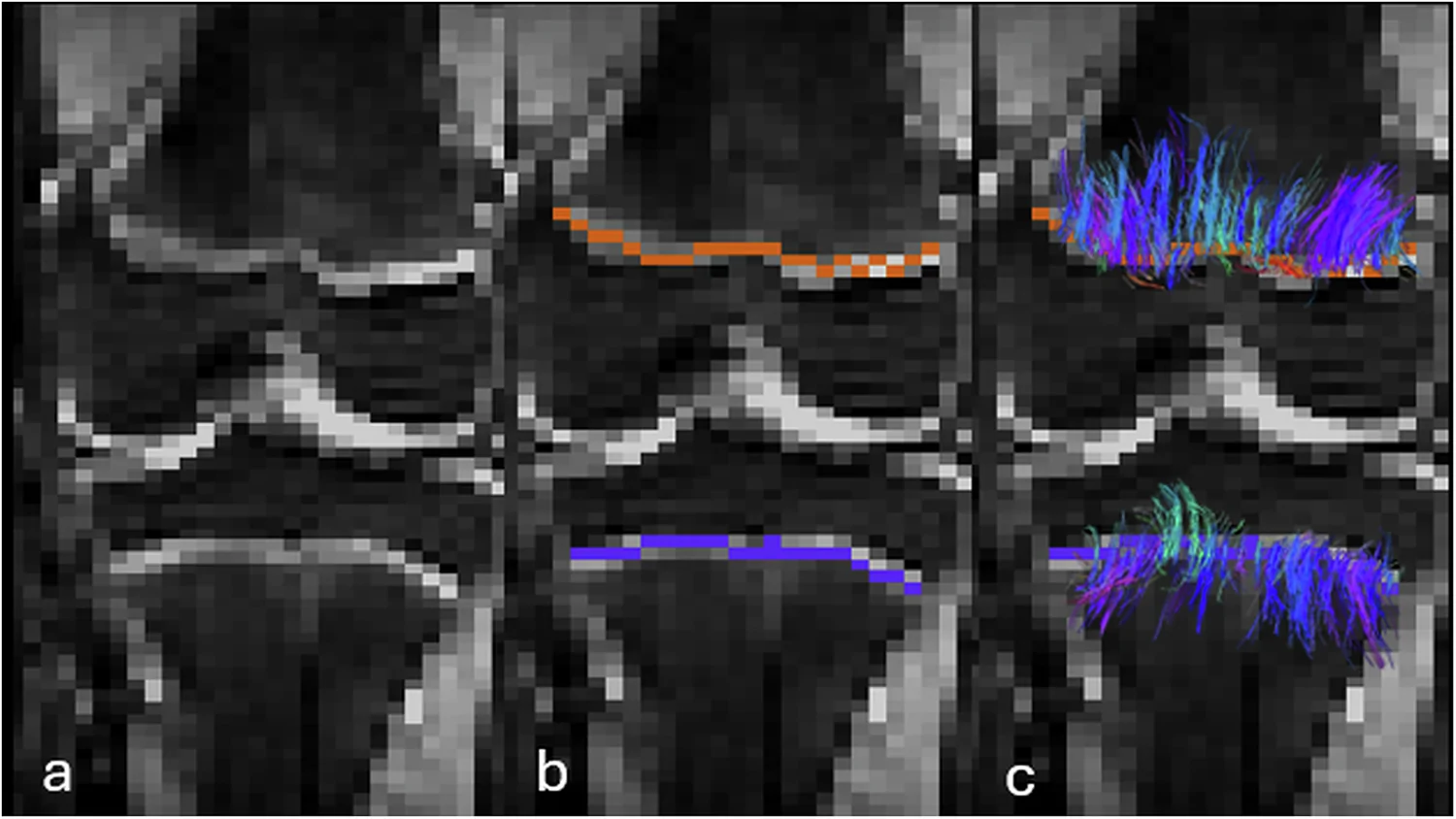
Manual segmentation on the b₀ image and resulting tractography reconstruction. Coronal *b* = 0 images, reformatted from sagittal acqusition, from a representative study subject. **a** Baseline b₀ image of the knee, showing the distal femoral physis (top) and proximal tibial physis (bottom). **b** The same image with manual segmentations of the distal femoral physis (orange) and proximal tibial physis (blue) overlaid. **c** The same image with segmentation overlays and the resulting deterministic tractography reconstruction; tract color encodes the principal diffusion direction. Both physes were segmented

Physeal regions were manually segmented on the b₀ image in ITK-SNAP (v3.8) by a postdoctoral researcher with more than one year of experience with segmentation and subsequently verified by two senior pediatric radiologists with 7 and 14 years of experience in DTI research, respectively, to ensure anatomical accuracy. Segmentation followed the brightest-voxel metric described in the literature for physeal cartilage identification [5], focusing on two regions of interest: the distal femoral physis and the proximal tibial physis. For this analysis, only the distal femoral physis was evaluated. Representative segmentations and tractography are shown in Fig. 1.

### Tractography processing

Diffusion tensor fitting was performed using Diffusion Toolkit to calculate voxel-wise FA and ADC.

Deterministic tractography was then conducted using Trackvis (Trackvis and Diffusion Toolkit v. 0.6.4, trackvis.org, Martinos Center for Biomedical Imaging, Boston, MA, USA) using a Fiber Assignment by Continuous Tracking–FACT algorithm. Ten FA threshold combinations were evaluated: minimum FA values of 0.05, 0.10, and 0.15 paired with maximum FA values of 0.40, 0.50, and 0.60, plus the original control threshold (FA_min = 0.15; FA_max = 1.0). The rationale for using lower thresholds was based on established knowledge that physeal cartilage demonstrates lower FA values (typically < 0.15), as described in animal studies by Wang et al, and on research by Kvist et al which demonstrated that tracts can persist even in closed physes when conventional threshold parameters are applied [14, 15].

For each threshold setting, several tractography metrics were extracted. Tract volume was defined as the total volume of voxels transected by reconstructed tracts; tract-weighted FA and ADC were computed as the mean FA and ADC values within those voxels.

### Statistical analysis

To assess the relationship between DTI metrics and height velocity, partial Pearson correlations were computed between baseline DTI metrics (tract volume, tract FA, and tract ADC) and height velocity, defined as the annualized change in height from baseline to the 12-month follow-up visit. Male and female participants were analyzed together to maximize statistical power. Tanner stage was included as a covariate in the partial correlation models to account for the confounding effects of pubertal stage. Furthermore, to evaluate the sensitivity of these associations to tractography parameter selection, we systematically varied the FA thresholds used during tract reconstruction. The magnitude of correlation coefficients was interpreted according to Cohen’s standard, where coefficients of 0.10, 0.30, and 0.50 represent small, medium, and large effect sizes, respectively [16].

For each threshold combination, partial correlations were calculated with 95% confidence intervals (CI) derived using Fisher’s z-transformation. The primary purpose of this analysis was to characterize how tractography-derived correlations behave across a range of FA threshold combinations rather than to evaluate the implications of a specific correlation. Reported *p*-values are therefore provided for descriptive purposes only; no adjustment for multiple comparisons was applied or considered necessary under this framing. All analyses were performed using Python 3.10 with SciPy 1.11. A Large Language Model (Google Gemini) was used solely for English language proofreading and formatting of text elements before submission.

## Results

**Fig. 2:**
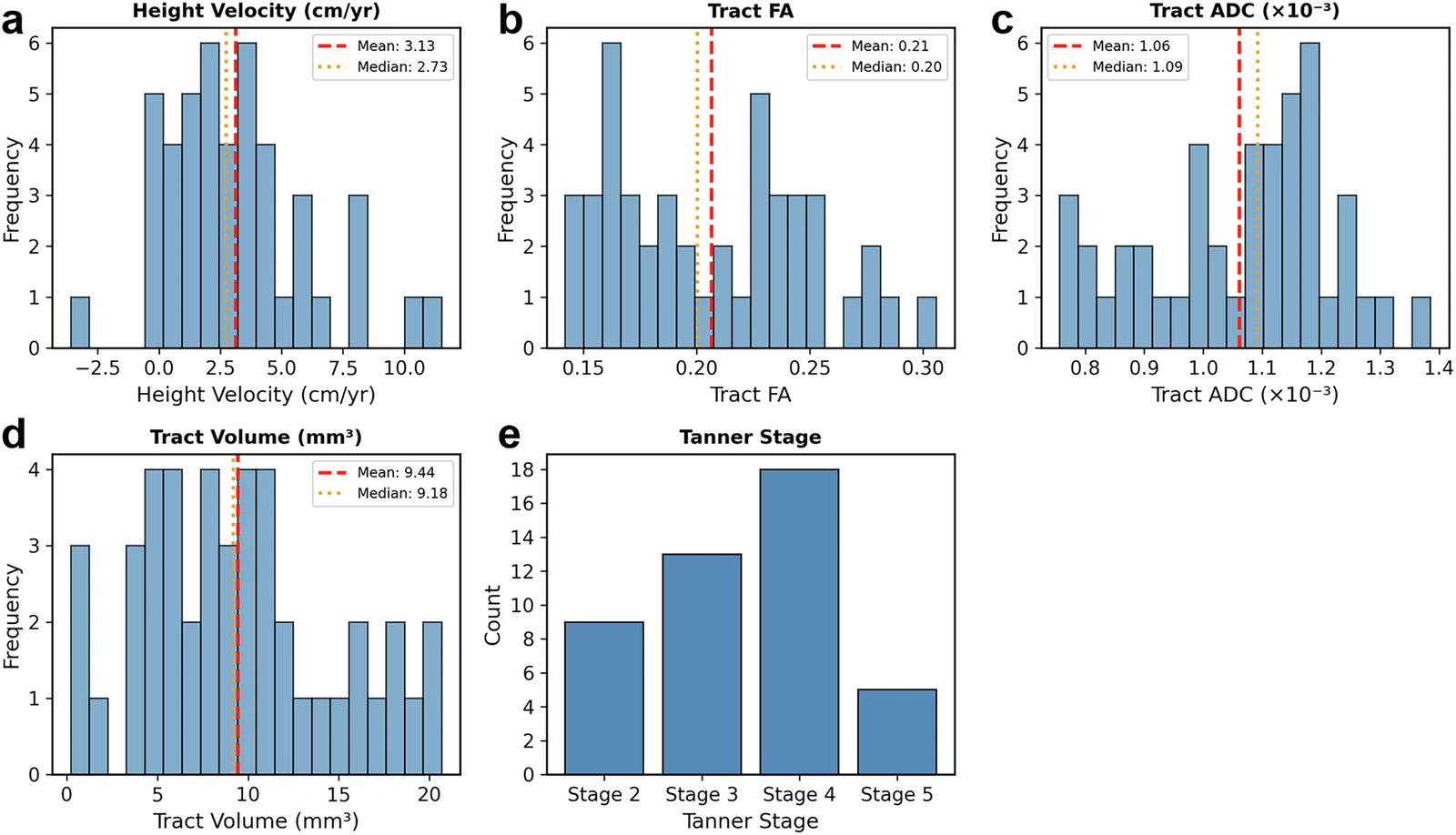
Distribution of study variables. Tract FA, tract ADC, and tract volume were derived using the conventional FA threshold (FA_min = 0.15; FA_max = 1.0) histograms showing the distribution of (**a**) annualized height velocity, (**b**) tract FA, (**c**) tract ADC, (**d**) tract volume, and (**e**) Tanner stage for the study cohort (*n* = 45). Red dashed lines indicate mean values; orange dotted lines indicate median values. ADC, Apparent diffusion coefficient; FA, Fractional anisotropy

The baseline characteristics of the study population showed an even distribution by sex, as well as by early and late stage of puberty, as summarized in Table 1, with the cohort distributions of all study variables shown in Fig. 2.

**Table 1:** Demographic characteristics of the study cohort

Total participants (*n* = 45)		
Age (years)	Mean (SD)	14.2 (1.3)
Sex	Female	25 (55.6%)
	Male	20 (44.4%)
Height (cm)	Mean (SD)	163.5 (10.0)
Weight (kg)	Mean (SD)	55.2 (15.3)
Tanner	Early pubertal (2–3)	22 (48.9%)
	Late pubertal (4–5)	23 (51.1%)
Height velocity (cm/y)	Mean (SD)	3.13 (2.97)

Data are presented as *n* (%) for categorical variables (sex, Tanner stage) and mean ± SD for all continuous variables

*SD* Standard deviation

### Tract ADC

**Fig. 3:**
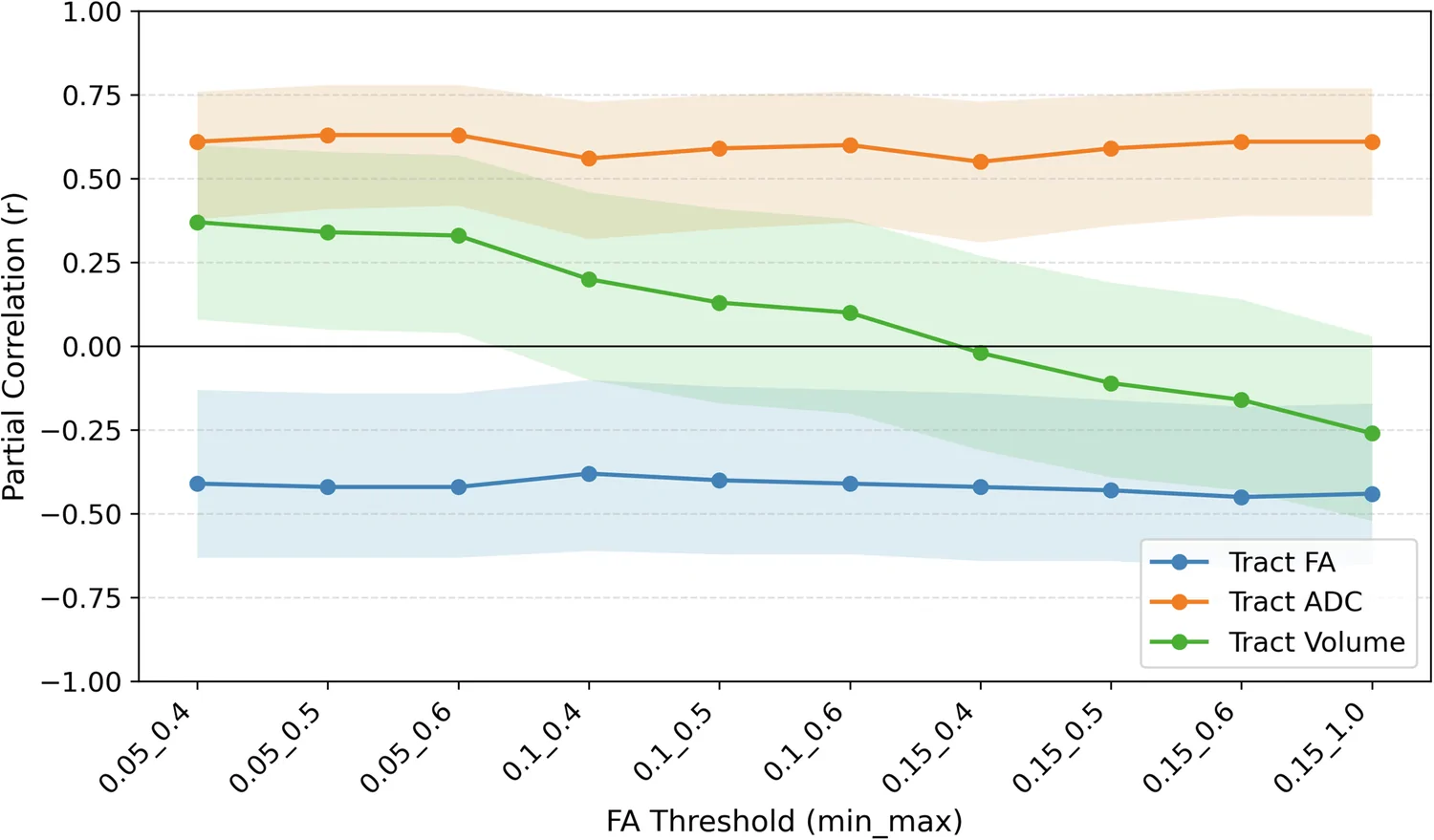
Sensitivity of DTI–growth plate associations to FA threshold selection. Partial Pearson correlations between PMC tractography metrics and annualized height velocity (*n* = 45) across 10 FA threshold combinations, controlling for pubertal status (Tanner stage). Solid lines connect point estimates; shaded bands show 95% CIs derived from Fisher *z*-transformation. Tract ADC (orange) and tract FA (blue) show stable correlations across thresholds, with confidence bands that do not cross zero, indicating significance at all tested thresholds. Tract volume (green) demonstrates threshold-dependent behavior—the 95% confidence band sits above zero at the lowest FA_min thresholds (0.05) and crosses zero as the minimum threshold is increased, reflecting loss of significance at conventional thresholds. ADC, Apparent diffusion coefficient; FA, Fractional anisotropy; FA_min, Minimum fractional anisotropy; PMC, Physeal–metaphyseal complex

When accounting for developmental status, partial correlations adjusted for Tanner stage revealed that tract ADC of the distal femoral PMC demonstrated consistently strong positive correlations with annualized height velocity across all ten FA threshold combinations tested (Fig. 3 and Supplementary Tables S1a–c). Nominal positive correlations were observed across all thresholds (*r* = 0.55–0.63; all nominal *p* < 0.001). The largest tract ADC–growth association was observed at an FA threshold of 0.05–0.60 (*r* = 0.63, 95% CI: [0.42, 0.78], nominal *p* < 0.001). The magnitude of these correlations was consistent across threshold variations: increasing FA min from 0.05 to 0.15 produced modest attenuation (Δ*r* ≈ 0.05), while varying FA_max from 0.40 to 1.00 had minimal effect.

### Tract FA

Tract FA showed consistent negative partial correlations with height velocity across all threshold combinations (*r* = -0.38 to -0.45). The largest tract FA–growth association was observed at FA threshold 0.15–0.60 (*r* = -0.45, 95% CI: [-0.66, -0.18], nominal *p* = 0.002).

### Tract volume

Tract volume associations with height velocity were highly sensitive to threshold selection (Fig. 3). At permissive FA thresholds (FA_min = 0.05), tract volume showed nominal positive correlations with height velocity: FA 0.05–0.40, *r* = 0.37 (95% CI: [0.08, 0.60], nominal *p* = 0.013); FA 0.05–0.50, *r* = 0.34 (95% CI: [0.05, 0.58], nominal *p* = 0.021); FA 0.05–0.60, *r* = 0.33 (95% CI: [0.04, 0.57], nominal *p* = 0.026).

As the minimum FA threshold was increased to an intermediate level (FA_min = 0.10), these partial correlations were attenuated and lost nominal significance (*r* = 0.10–0.20).

At more restrictive FA thresholds (FA_min = 0.15), the correlation coefficients shifted toward negative values but did not reach statistical significance, including the conventional threshold (FA 0.15–1.00), which yielded *r* = -0.26 (95% CI: [-0.52, 0.03]).

**Fig. 4:**
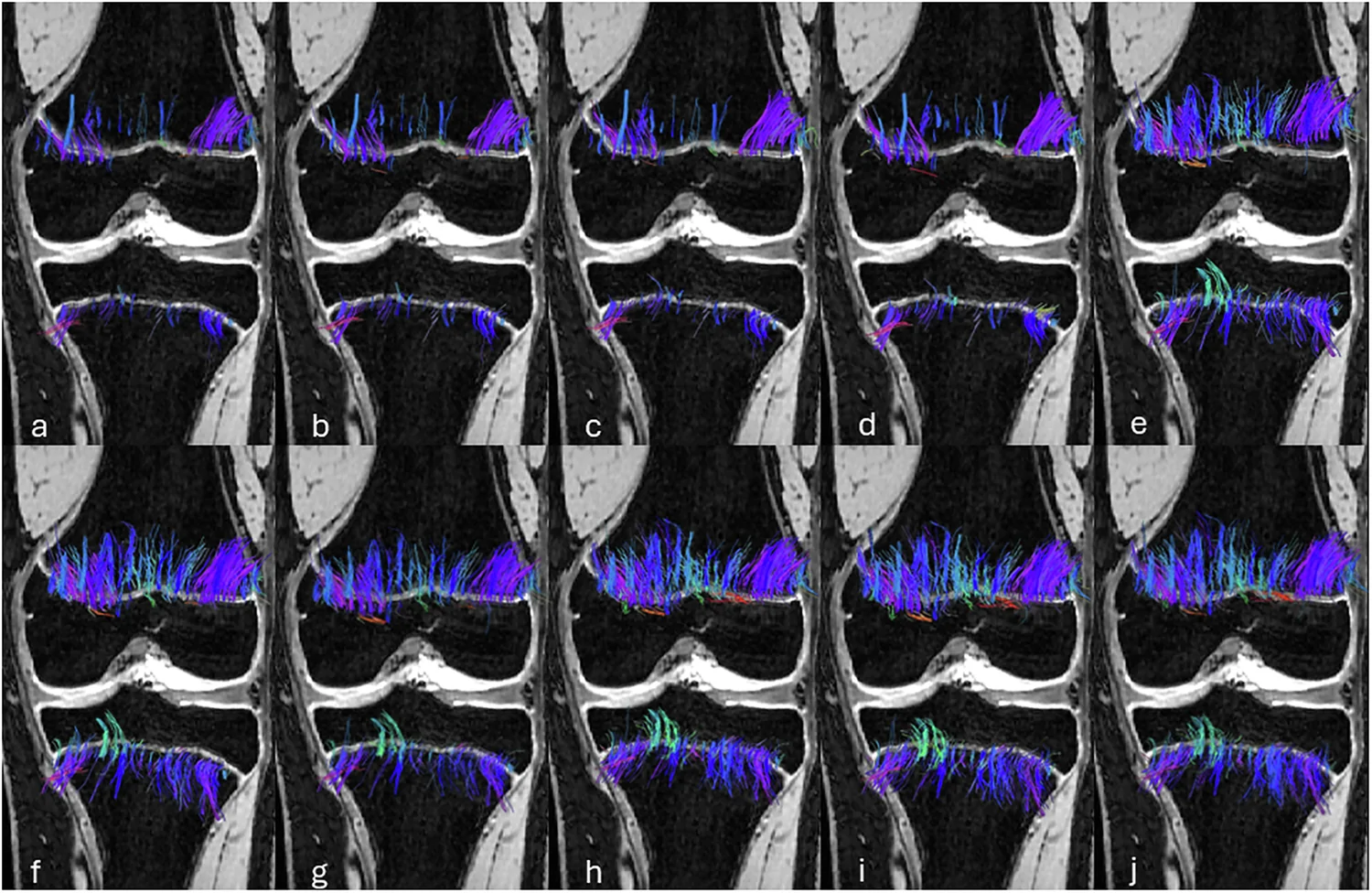
Tractography reconstruction across FA thresholds. Coronal reformatted gradient echo image of the distal femur with overlaid tractography reconstruction for a representative subject (early pubertal; height velocity 8.3 cm/year) across all 10 FA threshold combinations. **a** FA 0.15–0.4 = 6.1 mm³. **b** FA 0.15–0.5 = 6.2 mm³. **c** FA 0.15–0.6 = 6.3 mm³. **d** FA 0.15–1 = 6.3 mm³. **e** FA 0.1–0.5 = 18.8 mm³. **f** FA 0.1–0.4 = 18.8 mm³. **g** FA 0.1–0.6 = 18.9 mm³. **h** FA 0.05–0.5 = 25.3 mm³. **i** FA 0.05–0.6 = 25.3 mm³. **j** FA 0.05–0.4 = 25.5 mm³. Lower FA-min thresholds (**h**–**j**) capture a broader region of the PMC, including areas with less organized collagen architecture, resulting in larger tract volumes. FA, Fractional anisotropy; mm³, Cubic millimeters; PMC, Physeal–metaphyseal complex

This threshold-dependent pattern is qualitatively illustrated in Fig. 4. In a representative subject with high growth velocity (8.3 cm/year, early-pubertal) at the permissive threshold (FA 0.05–0.40), the tract volume was 25.5 mm³, whereas the restrictive conventional threshold (FA 0.15–1.0) yielded only 6.1 mm³.

## Discussion

Our study demonstrated that PMC diffusivity metrics are associated with height velocity regardless of FA threshold selection. Tract FA consistently decreased with height velocity across all thresholds tested [1, 14]. However, only the lower FA thresholds yielded nominally significant correlations between tract volume and height velocity, suggesting that selecting smaller minimum and maximum thresholds may capture the more diffuse, growth-sensitive aspects of the tracts and exclude tissue structures that may be less reflective of active longitudinal growth.

The relationship between DTI metrics and height velocity observed in the current study is complementary to findings previously reported in a rabbit model [14]. In that experimental study, FA increased with age, while ADC decreased. In our cohort, lower FA values and higher ADC values were associated with greater height velocity. This suggests that PMC tissue during periods of more rapid growth exhibits a less restricted microstructural environment, where a relative decrease in barrier structures—such as mineralized septa and dense collagen packing—allows for greater mean water diffusivity. Higher ADC may further reflect increased hydration, greater extracellular matrix turnover, or enlarged hypertrophic chondrocyte zones characteristic of rapid longitudinal growth.

DTI metrics, particularly ADC, demonstrated that greater diffusivity was associated with increased height velocity. Recent evidence suggests an association between diffusion metrics and skeletal maturation. Raya et al demonstrated that the orientation of the first eigenvector (axial diffusivity) aligns strongly with collagen fiber organization across cartilage zones, implying specificity to structural components such as type II collagen [17]. Moreover, comparative analysis of MRI-DTI with histology and micro-computed tomography (μCT) in rabbit growth plates indicates that elevated ADC reflects diffusion within the transitional (or “comb-like”) zone, characterized by ossified extracellular matrix interspersed with hypertrophic chondrocyte columns [14].

A clear difference in sensitivity to height velocity emerged between the thresholding methods. Specifically, at the lower thresholds (FA 0.05–0.4), tract volume was significantly associated with height velocity. In contrast, tract volume derived from the higher thresholds showed no significant correlation with height velocity, suggesting reduced sensitivity to dynamic growth processes. The lack of significant correlation is of interest since prior studies found that the number of tracts and tract volume were highest at skeletal maturation stage 4b, indicating that tractography could be sensitive to PMC activity [1].

Based on these findings, we suggest lower thresholds (FA_min: 0.05 and FA_max: 0.40) as starting points for optimization in future studies assessing PMC tract volume. Lower thresholds appear better positioned to capture the less organized hypertrophic and transitional zones linked to longitudinal growth, which are systematically underestimated by conventional FA thresholds. Given the descriptive, methodological focus of the present analysis, investigators should consider reporting results across multiple threshold combinations and validating their findings in larger cohorts powered specifically to estimate the underlying correlations before adopting any single threshold as a clinical standard.

A primary concern with permissive tractography thresholds, such as an FA_min of 0.05, is the potential inclusion of isotropic ‘noise’ leading to false-positive reconstructions. While higher thresholds (0.15–0.20) are standard in DTI to ensure specificity, they may be overly restrictive for tissues with lower baseline anisotropy [18]. For example, a study of the brachial plexus demonstrated that increasing the FA_min from 0.05 to 0.10 resulted in a 20% reduction in detected tracts, suggesting that standard thresholds significantly underestimate peripheral or transitional structures [19]. In the pediatric physis, our descriptive findings show that a 0.05 threshold is well positioned to potentially capture low-anisotropic microstructures and architecture that correspond to the transitional and hypertrophic zones. The significant correlation between tract volume and height velocity at FA_min = 0.05 (*r* = 0.37), which was lost at higher thresholds, suggests this additional volume represents a meaningful biological signal rather than stochastic noise.

Restricting the upper bound on FA (FA_max) is equally important for biasing the reconstruction toward growth-sensitive tissue components. Our prior work in adolescents and young adults [1] and in a rabbit growth-plate model [14] demonstrated that physeal FA rises toward skeletal maturation: as physeal closure approaches, the residual physis is increasingly dominated by mineralized, highly organized matrix, while the population of actively dividing chondrocyte columns shrinks. Retaining the highest-anisotropy voxels (FA up to 1.0) therefore preferentially samples this structurally mature tissue rather than the growth-active proliferative and hypertrophic zones. In parallel, age-related depletion of resting zone chondrocytes—a relatively isotropic chondrocyte population—has been documented as part of normal growth plate senescence [20]; this depletion could itself elevate the mean FA of the residual physis without reflecting more active growth and may in fact indicate diminished growth potential. Restricting FA_max therefore helps ensure that reconstructed tract volume is biased toward components reflecting growth activity rather than residual mineralized columns or post-resting-zone artefacts.

While this study describes an inclusive FA_min of 0.05 as the threshold at which growth-related tract volume is best preserved in this cohort, future research might explore a dual-threshold approach. Segmenting the PMC into low-FA (0.05–0.15) and high-FA (0.15–0.60) regions could potentially differentiate between the hypertrophic zones and the more organized proliferative zones. Furthermore, the strong correlations observed between ADC and height velocity reinforce the utility of the ADC map as a robust marker of physeal maturation and biological age. The cohort studied here spanned ages 12–17 years, so the optimal FA threshold identified in this analysis may require recalibration when applied to younger children, peripubertal cohorts, or cohorts in whom growth has decelerated; multi-center studies covering a broader range of skeletal maturation will be needed to establish the clinical robustness of these parameters across age groups.

Our study was limited by a small sample size. Imaging was acquired on two different vendor platforms (3-T GE Signa Premier and 3-T Siemens Magnetom Prisma) using an acquisition protocol where key parameters most directly affecting diffusion quantification (voxel size, *b*-values, diffusion direction scheme, and spatial coverage) were harmonized across sites. Although hardware differences required independent optimization of repetition and echo times across platforms rather than identical matching, this design minimizes inter-vendor variability, residual cross-platform bias cannot be entirely excluded in the absence of a traveling-subject calibration study. Future multi-center studies using fully harmonized or cross-validated protocols will be essential to confirm the robustness and applicability of these results in broader clinical and research settings. Other potential limitations include the narrow age range (early-to-mid adolescence), so findings may not generalize to younger children; future studies should aim to compare these threshold settings in younger and older populations. This study is positioned as a methodological description of how tractography-derived correlations behave across FA threshold combinations. The *p*-values reported in the figures and tables are therefore descriptive; no adjustment for multiple comparisons is applied because no hypothesis test about the underlying correlation structure is being made. By the same reasoning, the sample size (*n* = 45) is appropriate to demonstrate the methodological behavior under study but is not powered to estimate population-level effect sizes. Future studies seeking inferential estimates of the correlations between PMC tractography metrics and growth velocity in clinical populations should perform a priori power calculations to ensure adequate sample size.

Our study benefits from a longitudinal design in which each participant was scanned with a consistent acquisition protocol across time points. Such within-subject technical consistency is important for evaluating the sensitivity of FA thresholds and their effect on the correlation between DTI-derived tractography metrics and annualized height velocity.

PMC DTI metrics are significantly influenced by tractography parameter selection. While ADC and FA remain robust across various thresholds, tract volume associations with height velocity are attenuated by conventional FA thresholds and are best preserved at a permissive FA_min of 0.05 and FA_max of 0.40 in our cohort. The conventional thresholds of 0.15 and 1.0 appear less sensitive to growth-associated low-anisotropy PMC components, likely excluding the lower-anisotropy transitional zones critical to skeletal development and capturing tissues that are not part of the physis. These descriptive methodological findings support standardized reporting of tractography parameters as a prerequisite for developing reproducible DTI biomarkers in pediatric musculoskeletal imaging; FA_min of 0.05 and FA_max of 0.40 are proposed as starting points for optimization rather than a definitive clinical standard, pending inferential confirmation in larger cohorts powered specifically to estimate these correlations.

## Supplementary information

**Additional file 1: Table S1a** Partial correlations between DTI metrics and height velocity at the FA_min = 0.05 threshold family. **Table S1b** Partial correlations between DTI metrics and height velocity at the FA_min = 0.10 threshold family. **Table S1c** Partial Correlations between DTI metrics and height velocity at the FA_min = 0.15 threshold family.

## Data Availability

The datasets used and/or analyzed during the current study are available from the corresponding author on reasonable request.

## Abbreviations

ADC: Apparent diffusion coefficient
CI: Confidence interval
DTI: Diffusion tensor imaging
FA: Fractional anisotropy
MRI: Magnetic resonance imaging
PMC: Physeal–metaphyseal complex
