# Supplementary material for: Sensitivity of physeal-metaphyseal tractography correlations with height velocity for fractional anisotropy threshold selection
Source: Eur Radiol Exp. 2026 Oct 23;10:161. doi: 10.1186/s41747-026-00826-8 (PMC13694605; doi:10.1186/s41747-026-00826-8)
Supplement: Supplementary file 1 [file 41747_2026_826_MOESM1_ESM.pdf]

Sensitivity of Physeal-Metaphyseal Tractography Correlations with Height Velocity for Fractional Anisotropy Threshold selection

Electronic supplementary material

Table S1a Partial correlations between DTI metrics and height velocity at the FA_min = 0.05 threshold family

FA threshold	*Metric*	*r*	*95%* confidence interval	*p*-value
0.05–0.40	Tract FA	−0.41	[−0.63, −0.13]	0.005
	Tract ADC	0.61	[0.38, 0.76]	< 0.001
	Tract volume	0.37	[0.08, 0.60]	0.013
0.05–0.50	Tract FA	−0.42	[−0.63, −0.14]	0.005
	Tract ADC	0.63	[0.41, 0.78]	< 0.001
	Tract volume	0.34	[0.05, 0.58]	0.021
0.05–0.60	Tract FA	−0.42	[−0.63, −0.14]	0.004
	Tract ADC	0.63	[0.42, 0.78]	< 0.001
	Tract volume	0.33	[0.04, 0.57]	0.026

Table S1b Partial correlations between DTI metrics and height velocity at the FA_min = 0.10 threshold family

FA threshold	*Metric*	*r*	*95%* confidence interval	*p*-value
0.10–0.40	Tract FA	−0.38	[−0.61, −0.10]	0.009
	Tract ADC	0.56	[0.32, 0.73]	< 0.001
	Tract volume	0.20	[−0.10, 0.46]	0.196
0.10–0.50	Tract FA	−0.40	[−0.62, −0.12]	0.006
	Tract ADC	0.59	[0.35, 0.75]	< 0.001
	Tract volume	0.13	[−0.17, 0.41]	0.391
0.10–0.60	Tract FA	−0.41	[−0.62, −0.13]	0.006

	Tract ADC	0.60	[0.37, 0.76]	< 0.001
	Tract volume	0.10	[−0.20, 0.38]	0.526

Table S1c Partial Correlations between DTI metrics and height velocity at the FA_min = 0.15 threshold family

FA threshold	*Metric*	*r*	*95%* confidence interval	*p*-value
0.15–0.40	Tract FA	−0.42	[−0.64, −0.14]	0.004
	Tract ADC	0.55	[0.31, 0.73]	< 0.001
	Tract volume	−0.02	[−0.31, 0.27]	0.878
0.15–0.50	Tract FA	−0.43	[−0.64, −0.16]	0.003
	Tract ADC	0.59	[0.36, 0.75]	< 0.001
	Tract volume	−0.11	[−0.39, 0.19]	0.461
0.15–0.60	Tract FA	−0.45	[−0.66, −0.18]	0.002
	Tract ADC	0.61	[0.39, 0.77]	< 0.001
	Tract volume	−0.16	[−0.43, 0.14]	0.293
*0.15–1.00**	Tract FA	−0.44	[−0.65, −0.17]	0.003
	Tract ADC	0.61	[0.39, 0.77]	<0.001
	Tract volume	−0.26	[−0.52, 0.03]	0.083

In these three tables, we represent partial Pearson correlations between PMC tractography metrics and annualized height velocity, controlling for pubertal status (Tanner stage; *n* = 45). Ten FA threshold combinations were evaluated FA_min–FA_max. *Original/control threshold parameters. *ADC* Apparent diffusion coefficient, *DTI* Diffusion tensor imaging, *FA* Fractional anisotropy, *PMC* Physeal-metaphyseal complex.
